# Machine learning for prediction of in-hospital mortality in lung cancer patients admitted to intensive care unit

**DOI:** 10.1371/journal.pone.0280606

**Published:** 2023-01-26

**Authors:** Tianzhi Huang, Dejin Le, Lili Yuan, Shoujia Xu, Xiulan Peng

**Affiliations:** 1 Department of Rehabilitation, The Second Affiliated Hospital of Jianghan University, Wuhan, China; 2 Department of Respiratory Medicine, People’s Hospital of Daye, The Second Affiliated Hospital of Hubei Polytechnic University, Daye, Hubei, China; 3 Department of Anesthesiology, The Second Affiliated Hospital of Jianghan University, Wuhan, China; 4 Department of Orthopedics, Sinopharm Dongfeng General Hospital, Hubei University of Medicine, Shiyan, Hubei, China; 5 Department of Oncology, The Second Affiliated Hospital of Jianghan University, Wuhan, China; Clinica Luganese Moncucco, SWITZERLAND

## Abstract

**Backgrounds:**

The in-hospital mortality in lung cancer patients admitted to intensive care unit (ICU) is extremely high. This study intended to adopt machine learning algorithm models to predict in-hospital mortality of critically ill lung cancer for providing relative information in clinical decision-making.

**Methods:**

Data were extracted from the Medical Information Mart for Intensive Care-IV (MIMIC-IV) for a training cohort and data extracted from the Medical Information Mart for eICU Collaborative Research Database (eICU-CRD) database for a validation cohort. Logistic regression, random forest, decision tree, light gradient boosting machine (LightGBM), eXtreme gradient boosting (XGBoost), and an ensemble (random forest+LightGBM+XGBoost) model were used for prediction of in-hospital mortality and important feature extraction. The AUC (area under receiver operating curve), accuracy, F1 score and recall were used to evaluate the predictive performance of each model. Shapley Additive exPlanations (SHAP) values were calculated to evaluate feature importance of each feature.

**Results:**

Overall, there were 653 (24.8%) in-hospital mortality in the training cohort, and 523 (21.7%) in-hospital mortality in the validation cohort. Among the six machine learning models, the ensemble model achieved the best performance. The top 5 most influential features were the sequential organ failure assessment (SOFA) score, albumin, the oxford acute severity of illness score (OASIS) score, anion gap and bilirubin in random forest and XGBoost model. The SHAP summary plot was used to illustrate the positive or negative effects of the top 15 features attributed to the XGBoost model.

**Conclusion:**

The ensemble model performed best and might be applied to forecast in-hospital mortality of critically ill lung cancer patients, and the SOFA score was the most important feature in all models. These results might offer valuable and significant reference for ICU clinicians’ decision-making in advance.

## Introduction

Lung cancer is the third most common malignancy and is reported the leading cause of cancer death in males and the second most common cancer in females, which taking up more than one-fifth of all cancer deaths worldwide [1–3]. Exceed 158,000 patients died from lung cancer in the United States in 2016, which accounted for 27% of all cancer deaths [4, 5], the prognosis remains poor although improvement has been made in the therapy of lung cancer, the 5-year survival rate for all stages combined is only 15% [6, 7]. Many lung cancer patients require admitted to intensive care unit (ICU) and respiratory failure requiring mechanical ventilation is the major reason for lung cancer patients being admitted to the ICU [8, 9]. Although progressive improvement has been made to improve the prognosis in lung cancer patients admitted to the ICUs, the mortality rate remains extremely high, the mortality rate in lung cancer patients admitted to ICU was 43% and the in-hospital mortality is 60%, and the mortality rate is higher in patients with stage IV (68%) [10]. Currently, the lack of early prediction and risk stratification for in-hospital mortality is the main challenge for ICU clinicians. The decision regarding which groups of lung cancer patients admitted to the ICU at high-risk and would have poor prognosis is based on a complex suite of considerations including therapeutic options and the wishes of patients and their family. These critically ill lung cancer patients usually have poor long-term survival and high financial cost. Hence, it’s necessary to explore risk prediction models to distinguish those at high-risk of critically ill lung cancer patients admitted to ICU.

The development of artificial intelligence has led to a significant improvement in the predictive models used for estimating the risk of mortality in cancer patients. Machine learning (ML), a new type of artificial intelligence can transform measurement results into relevant predictive models, especially cancer models, based on the rapid development of large datasets and deep learning. Recently, ML have been shown to be effective in predicting lung cancer susceptibility, recurrence, and survival of malignant tumors [11–13]. However, there is still limited data relating to the in-hospital mortality risk prediction models using ML methods in patients with lung cancer in the ICU setting.

Therefore, this study aimed to develop six ML algorithm models including logistic regression, decision tree, random forest, light gradient boosting machine (GBM), extreme gradient boosting (XGBoost), and an ensemble model to predict the in-hospital mortality among lung cancer patients admitted to ICU so that individual prevention strategies for critically ill lung cancer patients could be proposed to help clinicians to make therapeutic decisions. Moreover, we also intended to compared the six ML models and determined the best model for in-hospital mortality prediction in lung patients admitted to the ICU.

## Methods

### Data source

This retrospective study utilized information from the eICU Collaborative Research Database (eICU-CRD) [14] and the Medical Information Mart for Intensive Care-IV (MIMIC-IV version 1.0) database [15], eICU-CRD contains data of more than 200 thousand ICU admissions in 2014 and 2015 at 208 US hospitals while MIMIC-IV includes information of more than 70,000 patients admitted to the ICUs of Beth Israel Deaconess Medical Center in Boston, MA, from 2008 to 2019. Due to the data used in this study were extracted from public databases, it was exempt from the requirement for informed consent from patients and approval of the Institutional Review Board (IRB). All procedures were performed according to the ethical standards of the 1964 Helsinki Declaration and its later amendments or comparable ethical standards. After finishing the web-based training courses (S1 Fig) and the Protecting Human Research Participants examination, we obtained permission to extract data from the eICU-CRD and MIMIC-IV database.

### Cohort selection

**Fig 1:**
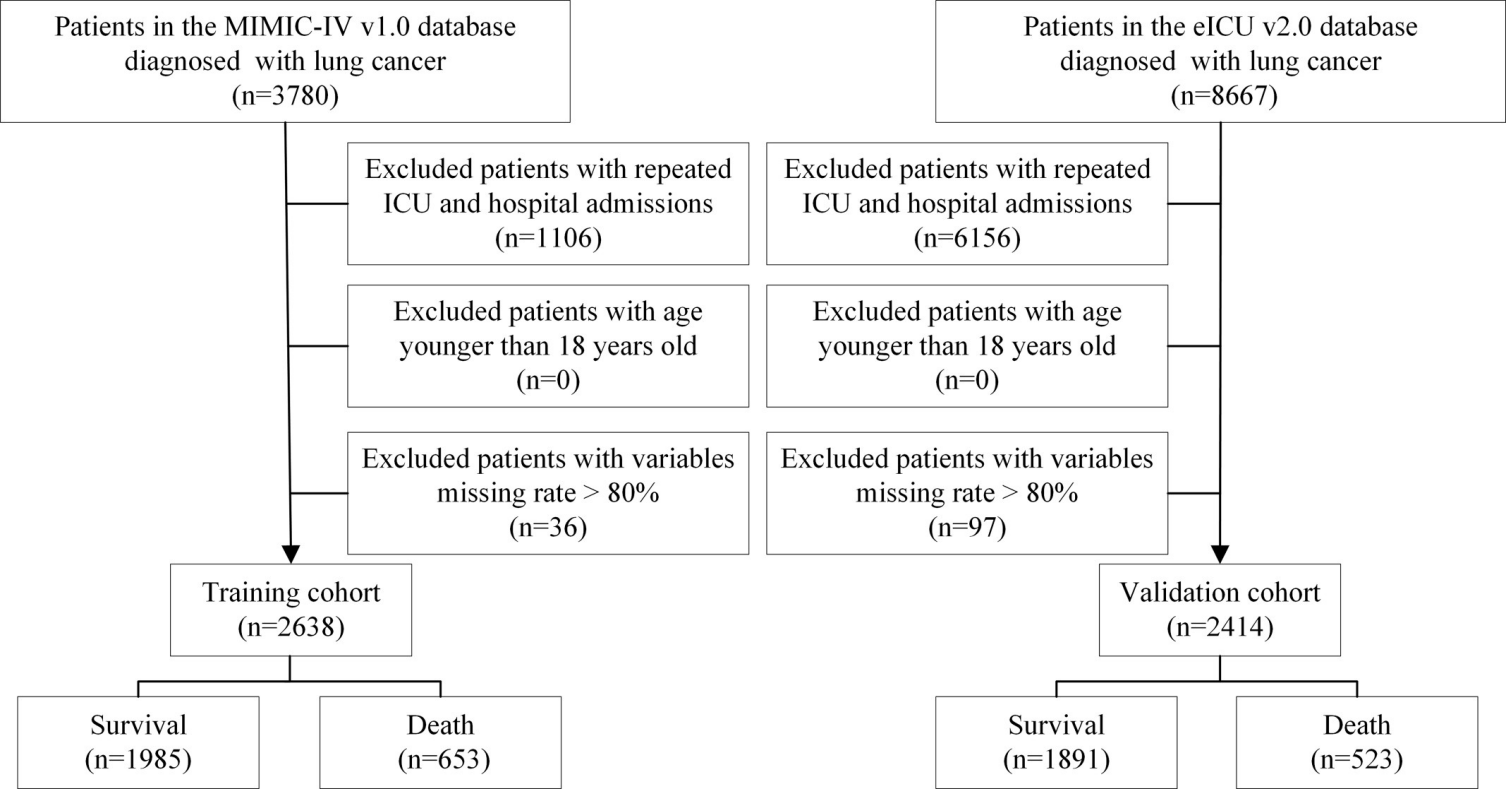
The flow chart of this study.

Patients with one of the following conditions were excluded: (1) less than 18-year-old at first admission to ICU; (2) repeated ICU admissions; (3) more than 80% of personal data was missing. We randomly selected MIMIC-IV database as the training cohort and eICU-CRD database as the validation cohort. A total of 2,638 patients in the MIMIC-IV database assigned into the training cohort and 2,414 patients in the eICU-CRD database assigned into the validation cohort were finally included in this study, the detailed flowchart was shown in Fig 1.

### Date collection and outcomes

Baseline characteristics and admission information: age, gender and body mass index (BMI)were calculated as described in previous studies. Comorbidities including hypertension, diabetes, chronic kidney disease, myocardial infarction, congestive heart failure, atrial fibrillation, valvular disease, chronic obstructive pulmonary disease, stroke, hyperlipidemia and liver disease were also collected for analysis based on the recorded ICD codes in the two databases. Charlson comorbidity index (CCI) was also included. In addition, severity scores including sequential organ failure assessment (SOFA) score, the oxford acute severity of illness score (OASIS), the acute physiology score III (APSII) were collected. Acute complications during ICU including acute heart failure, acute respiratory failure, acute hepatic failure and cardiac arrest based on ICD codes, acute kidney injury based on KDIGO guideline in 48 hours [16], sepsis based on sepsis 3.0 criteria [17] were also recorded. In addition, initial vital signs and laboratory results were also measured during the first 24 hours of ICU admission.

The primary outcome was in-hospital mortality.

### Statistical analysis

For all continuous covariates, the mean values and standard deviations are reported Categorical data were expressed as frequency (percentage). The Chi-square test or Fisher’s test was appropriately performed to compare the differences between groups. The baseline characteristics were reported as a training cohort and validation cohort. The comparison of baseline characteristics was performed in R software (version 4.1.0). *P* < 0.05 was considered statistically significant. Modeling work were done using Python 3.6.4.

### Construction of in-hospital mortality predictive models

Logistic regression, decision tree, random forest, and two gradient boosting decision trees, including LightGBM, and XGBoost, were adopted to construct prediction models. In order to improve prediction, an ensemble model was constructed, which applied staking strategy using random forest, LightGBM and XGBoost [18]. The prediction probabilities of the three models were input into a logistic regression model to produce a final prediction. Hence, six in-hospital mortality predictive models were developed using logistic regression, decision tree, random forest, LightGBM, XGBoost and ensemble models, which each used 100 full features for each time window. Furthermore, the top 10 important features derived from random forest, lightGBM, and XGBoost model were also analysis [18].

### Performance evaluation

To evaluate and compare the predictive accuracy of prediction by decision tree, random forest, LightGBM, XGBoost, ensemble model and logistic regression models. Each model was evaluated according to accuracy, recall, F1 score, and AUC (area under the receiver operating characteristic) curve [19].

### SHAP analysis

To further analyze the positive or negative effect of the important features identified for in-hospital mortality prediction and investigate the relationship between, a shapely additive explanations (SHAP) analysis was performed using Python 3.7.0. The SHAP value is the assigned predicted value of each feature of the data [20].

## Results

### Baseline characteristics

A total of 5,052 patients were finally included in the present study, including 2,638 patients in the training cohort extracted from the MIMIC-IV database and 2,414 patients in the validation cohort extracted from the eICU-CRD database. There were 653 (24.8%) in-hospital death in the training cohort, and 523 (21.7%) in-hospital death in the validation cohort. Table 1 showed the baseline characteristics both in the training cohort and in the validation cohort.

**Table 1:** Comparisons of baseline characteristics in all cohorts.

Characteristics	Training cohort	Validation cohort	P value
N	2638	2414	
Age, years old	68.9 ± 12.1	66.5 ± 11.3	<0.001
Gender, male, n (%)	1361 (51.6)	1272 (52.7)	0.451
BMI, kg/m^2^	26.1 ± 7.6	26.0 ± 7.3	0.138
Ethnicity, n (%)			<0.001
White	1924 (72.9)	1950 (80.8)	
Black	302 (11.4)	247 (10.2)	
Others	412 (14.7)	217 (9.0)	
Tumor type, n (%)			<0.001
Primary	1804 (68.4)	1889 (78.3)	
Metastatic	834 (31.6)	525 (21.7)	
Critical care procedure, n (%)			
Mechanical ventilation	901 (34.2)	738 (30.6)	0.007
Continuous renal replacement therapy	35 (1.3)	20 (0.8)	0.117
Vasopressors	718 (27.2)	359 (14.9)	<0.001
Comorbidities, n (%)			
Myocardial infarction	339 (12.9)	186 (7.7)	<0.001
Congestive heart failure	561 (21.3)	250 (10.4)	<0.001
Hypertension	1090 (41.3)	1223 (50.7)	<0.001
Diabetes	608 (23.0)	493 (20.4)	0.026
Chronic kidney disease	462 (17.5)	171 (7.1)	<0.001
Liver disease	198 (7.5)	21 (0.9)	<0.001
Chronic obstructive pulmonary disease	1034 (39.2)	796 (33.0)	<0.001
Stroke	246 (9.3)	127 (5.3)	<0.001
Atrial fibrillation	720 (27.3)	248 (10.3)	<0.001
Hyperlipidemia	888 (33.7)	102 (4.2)	<0.001
Charlson comorbidity index, points	9.4 (2.7)	6.9 (1.7)	<0.001
Acute complications during ICU, n (%)			
Acute heart failure	247 (9.4)	100 (4.1)	<0.001
Acute respiratory failure	765 (29.0)	904 (37.4)	<0.001
Acute hepatic failure	31 (1.2)	6 (0.2)	<0.001
Acute kidney injury	582 (22.1)	363 (15.0)	<0.001
Sepsis	1244 (47.2)	434 (18.0)	<0.001
Cardiac arrest	84 (3.2)	58 (2.4)	0.111
Score system, points			
Sequential organ failure assessment	4.4 ± 1.4	3.8 ± 1.3	<0.001
Oxford acute severity of illness score	32.3 ± 9.0)	24.8 ± 10.0	<0.001
Acute physiology score III	48.3 ± 12.8)	47.9 ± 21.5	0.594
Vital signs			
Systolic blood pressure, mmHg	123.7 ± 24.1	123.3 ± 24.5	0.507
Diastolic blood pressure, mmHg	69.4 ± 17.3	69.6 ± 16.4	0.661
Mean arterial pressure, mmHg	83.9 ± 17.9	83.7 ± 18.3	0.657
Heart rate, bpm	94.8 ± 21.6)	97.3 ± 21.8	<0.001
Respiratory rate, bpm	20.9 ± 6.6	21.3 ± 6.3	0.057
Temperature, °C	36.7 ± 0.7	36.8 ± 0.7	0.001
SpO2, %	96.1 ± 4.3	95.3 ± 6.0	<0.001
Laboratory values			
White blood cell, × 109/L	11.5 ± 8.7	12.2 ± 9.7	0.011
Hemoglobin, g/dL	10.7 ± 2.2	11.0 ± 2.3	<0.001
Platelet, × 109/L	251.2 ± 81.6	235.6 ± 71.4	<0.001
Hematocrit, %	32.7 ± 6.2	33.6 ± 6.8	<0.001
Mean corpuscular volume, fL	90.6 ± 7.7	90.3 ± 7.1	0.184
Mean corpuscular hemoglobin, pg	29.5 ± 2.9	29.5 ± 2.6	0.418
Mean corpusular hemoglobin concerntration, g/L	32.6 ± 1.7	32.8 ± 1.5	<0.001
Red blood cell, × 1012/L	3.6 ± 0.7	3.7 ± 0.8	<0.001
Red cell distribution width, %	15.7 ± 2.5	16.1 ± 2.6	<0.001
Albumin, g/dL	3.1 ± 0.7	3.0 ± 0.7	<0.001
Bilirubin, mmol/L	1.2 ± 0.4	0.9 ± 0.3	<0.001
Anion gap, mEq/L	14.8 ± 3.6	10.8 ± 4.0	<0.001
Bicarbonate, mEq/L	24.1 ± 4.7	25.8 ± 5.2	<0.001
Glucose, mg/dL	136.5 ± 54.3	141.4 ± 51.7	0.019
Blood urea nitrogen, mg/dL	24.3 ± 8.9	23.9 ± 8.6	0.378
Creatinine, mg/dL	1.2 ± 0.5	1.1 ± 0.4	0.457
Calcium, mg/dL	8.6 ± 1.0	8.7 ±1.0	<0.001
Chloride, mmol/L	101.7 ± 6.3	100.6 ± 6.5	<0.001
Potassium, mmol/L	4.3 ± 0.7	4.2 ± 0.7	<0.001
Sodium, mmol/L	137.4 ± 5.1	136.2 ± 5.5	<0.001
Prothrombin time, s	15.4 ± 5.2	15.6 ± 5.7	0.329
Activated partial thromboplastin time, s	35.2 ± 10.3	36.3 ± 11.1	0.062
International normalized ratio	1.4 ± 0.5	1.4 ± 0.4	0.500
Length of hospital, days	7.4 (4.3, 12.1)	6.9 (4.1, 11.7)	0.012
Length of ICU, days	2.0 (1.1, 4.0)	2.1 (1.1, 4.0)	0.490
Death, n (%)	653 (24.8)	523 (21.7)	0.010

### Model performance

**Fig 2:**
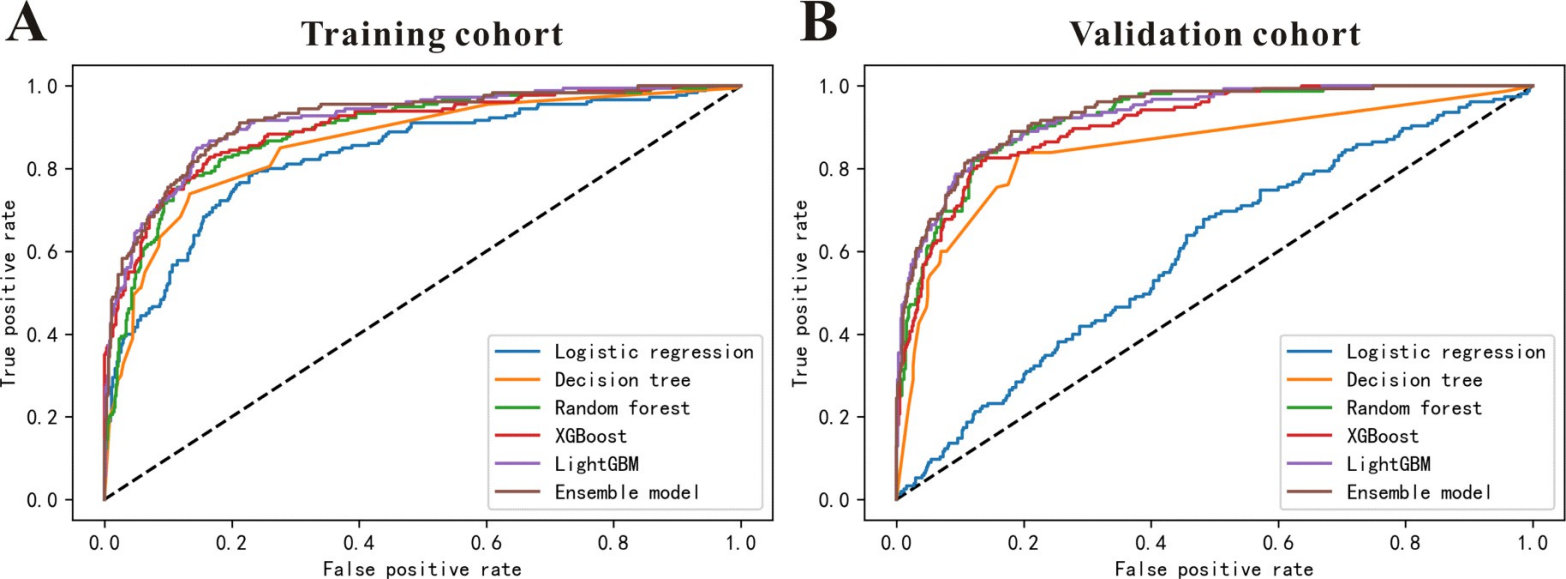
The performance of the six in-hospital mortality predictive models. ROC curves of the six prediction models using all features for predicting in-hospital mortality (**A**) in training cohort and (**B**) in the validation cohort.

Six models, logistic regression, decision tree, random forest, LightGBM, XGBoost, and ensemble models were used to predict in-hospital mortality using all the features. As can been seen in Table 2, the traditional model logistic regression exhibited the worst predictive ability, followed by decision tree, random forest, XGBoost, LightGBM. And the ensemble model showed the best predictive ability with the highest accuracy (0.89), recall (0.80), F1 score (0.82) and AUC (0.92) in training cohort. And the results in the validation cohort similar to the results in the training cohort (Table 2). In addition, we also performed ROC analysis to further confirm the in-hospital mortality predictive ability of these six models, as shown in Fig 2A and 2B, the logistic regression model depicted the worst predictive ability, followed by decision tree, random forest, XGBoost, LightGBM. And the ensemble model showed the best predictive performance both in the training cohort and in the validation cohort.

**Table 2:** Performance of the prediction models using all features.

Model	Accuracy	Recall	F1 score	AUC
Training cohort				
Logistic regression	0.83	0.68	0.71	0.83
Decision tree	0.85	0.73	0.75	0.86
Random forest	0.84	0.70	0.78	0.89
XGBoost	0.86	0.77	0.79	0.90
LightGBM	0.88	0.80	0.81	0.91
Ensemble model	0.89	0.80	0.82	0.92
Validation cohort				
Logistic regression	0.79	0.79	0.70	0.60
Decision tree	0.86	0.74	0.75	0.85
Random forest	0.84	0.84	0.80	0.92
XGBoost	0.87	0.87	0.77	0.91
LightGBM	0.88	0.80	0.82	0.92
Ensemble model	0.89	0.89	0.88	0.93

### Feature importance analysis

**Fig 3:**
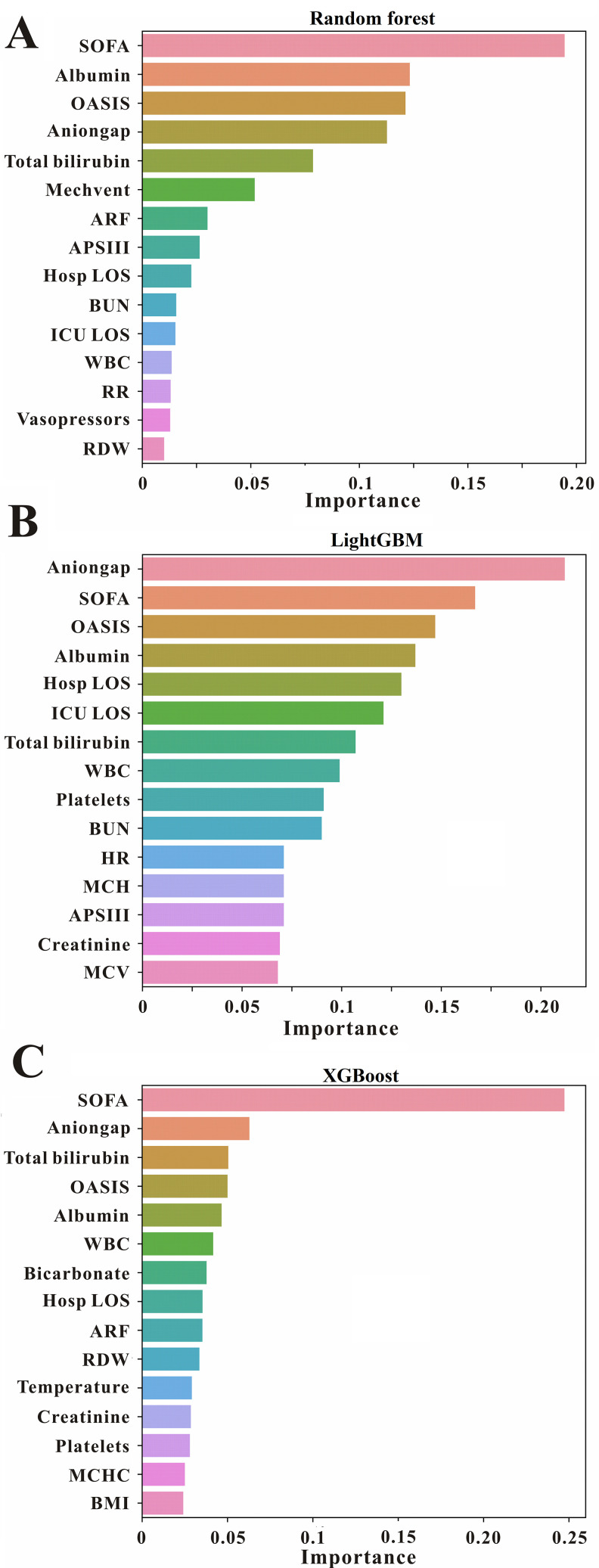
The important features of different models. The top 15 features derived from (**A**) random forest, (**B**) lightGBM, and (**C**) XGBoost model.

To clarify the important features that impacts on model output, the feature importance analysis was conducted. The top 15 features derived from random forest, lightGBM, and XGBoost model were shown in Fig 3. In random forest model, SOFA score was the most influential feature, followed by albumin, OASIS score, anion gap, billirubin, mechanical ventilation, acute respiratory failure, APSIII score, length of hospital, BUN, WBC, respiratory rate, vasopressors usage and RDW, and these features also had important on random forest model (Fig 3A). For lightGBM model, anion gap played the most important role in prediction in-hospital mortality, moreover, SOFA score, OASIS score, albumin, length of hospital, billirubin, WBC, platelet, BNU, heart rate, MCH, APSIII score, creatinine and MCV also plays important role in prediction (Fig 3B). Furthermore, in terms of XGBoost model, SOFA score had the most influence on in-hospital mortality prediction, followed by anion gap, billirubin, OASIS score, albumin, white blood cell, bicarbonate, length of hospital, acute respiratory failure, RDW, temperature, creatinine, platelet, MCHC and BMI (Fig 3C). Moreover, the feature importance analysis derived from random forest, lightGBM, and XGBoost model were also conducted in validation cohort in S2–S4 Figs. And the results were coincided with the result of the training cohort.

### SHAP analysis

**Fig 4:**
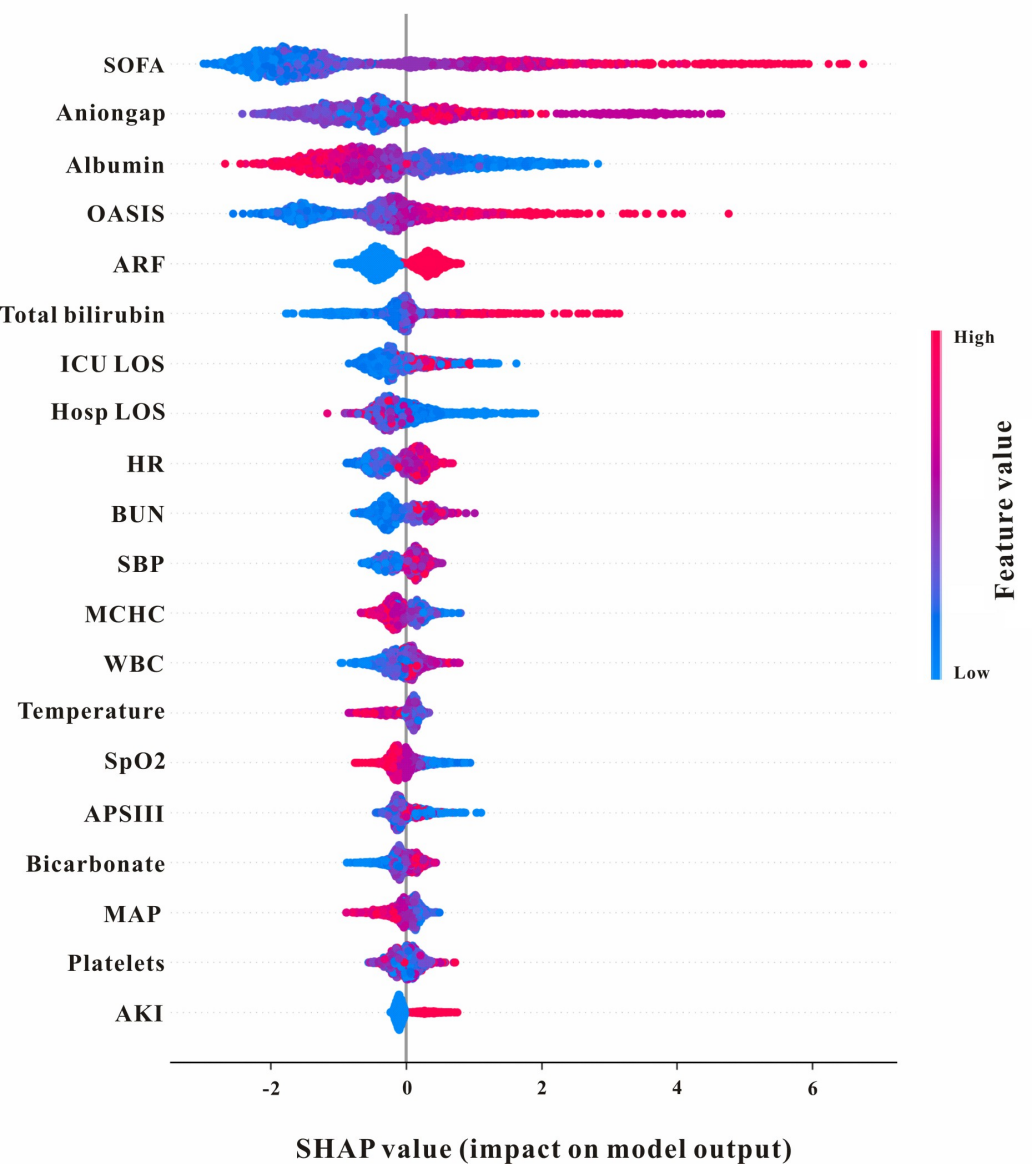
SHAP summary plot of the features of the XGBoost model. The higher the SHAP value of a feature, the higher the probability of in-hospital mortality development. A dot is created for each feature attribution value for the model of each patient, and thus one patient is allocated one dot on the line for each feature. Dots are colored according to the values of features for the respective patient and accumulate vertically to depict density. Red represents higher feature values, and blue represents lower feature values.

In order to manifest an overall positive or negative impact on model output, and to analyze the similarities and differences of important characteristics of critically ill lung cancer with different severities, the SHAP summary chart was used. As shown in Fig 4, SOFA score ranked the first in importance among the top 20 features of the XGBoost model, and the higher the SOFA score, the higher probability of in-hospital mortality development, indicating that SOFA score should be observed first in in-hospital mortality prediction.

**Fig 5:**
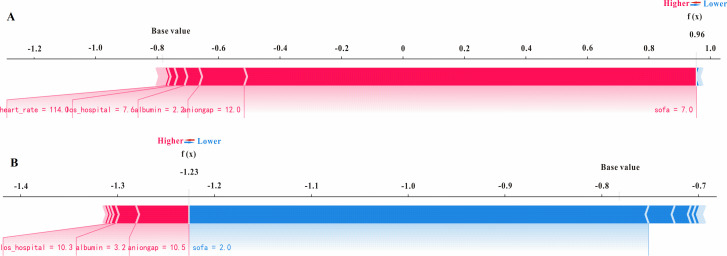
The SHAP force plots. The two representative SHAP force plots of a (**A**) dead and (**B**) survival patient. SHAP force plots are effective in interpreting the prediction value of the model in critical instances. The contribution of each feature to the output predicted value is shown with arrows with their force associated with the shapley values. Red arrows indicate features increasing the prediction results (i.e., yield values) to reach the predicted value (output value). Blue arrows show features decreasing the prediction values to reach the same output value. The arrows with positive and negative effects on yield values compensate on a point which is the prediction (output) value.

Taking the XGBoost model with excellent performance for predicting dead/survival using all features as an example, combined with the SHAP analysis method, a representative dead patient and a survival patient were selected to illustrate the effect of features on the prediction ability. As shown in Fig 5, for predicting dead patients, SOFA score plays a major positive role in the prediction results, the SHAP value of final model predicted for this patient is 0.96, which is beyond than 0, thus successfully predicting the patient as an in-hospital died patient. For predicting survival patients, anion gap plays a major positive role in the prediction results, SOFA score played a major negative role in predicting outcomes, the SHAP value of final model predicted for this patient is -1.23, which is less than 0, thus successfully predicting the survival patient.

## Discussion

In this retrospective study, we developed and validated machine learning algorithms based on clinical features based on largely public database MIMIC-IV and eICU-CRD, to predict in-hospital mortality of critically ill lung cancer patients. The lightGBM model exhibited the best performance for single model prediction, whereas the RF + ensemble model an ensemble model was constructed, which applied staking strategy using random forest, LightGBM and XGBoost exhibited the greatest AUC among the models we tested. Using advanced machine learning techniques, we could identify some important clinical features associated with in-hospital mortality such as SOFA score, anion gap, albumin, OASIS score and acute respiratory failure. These results have some implications and require further consideration.

ICU-related in-hospital mortality for lung cancer is ranked highest among the solid tumors and the in-hospital mortality in lung cancer patients admitted to ICU is discrepancy according to the lung cancer stage. Previous studies reported that the ICU mortality of extensive or advanced lung cancer patients over 50%. Park et al. investigated patients in Korea who had been newly diagnosed with lung cancer between 2008 and 2010 and indicated that the in-hospital mortality was 58.3% in those advanced critically ill lung cancer patients [21]. In addition, Song et al. analyzed the advanced lung cancer patients, including stage IIIB or IV non-small cell lung cancer and extensive-stage small cell lung cancer, admitted to the ICU and found before and after 2011, the in-hospital mortality was 82.4% and 65.9% [22]. In this study, our result manifested a similar result to Adam et al. [23] report a 20% in-hospital mortality rate in stage I non-small cell lung cancer. This maybe due to the vast majority of the type of the lung cancer were primary but not metastatic, so the in-hospital mortality in the present study is lower than those with advanced critically ill lung cancer patients. Unfortunately, it is difficult for clinicians to identify patients at high risk of in-hospital death in the ICU. Therefore, developing and promoting reliable prediction models is particularly urgent for identifying these patients and providing them with timely and effective interventions to improve their prognosis.

Currently, given the increasing applicability and effectiveness of supervised machine learning algorithms in predictive disease modeling, the breadth of research seems to progress [24, 25]. The well-known supervised learning classifiers, including support vector machine, random forest, convolutional neural network, and decision tree, have been gradually applied to clinical practice [26, 27]. With the help of machine learning classification, it showed that the machine learning-assisted decision-support model has more advantages than the traditional linear regression model. In this study, we used six different machine learning methods (logistic regression, decision tree, random forest, LightGBM, XGBoost, and ensemble models) to build predictive models. Four popular metrics (ROC, F1 score, accuracy and recall) were used to evaluate the performance of these algorithms. There is no doubt that the results showed that the ensemble model (which combined random forest, LightGBM and XGBoost) achieved the best performance and predictive stability, which was consistent with previous reported [18]. Apart from this, lightGBM model achieved the best predictive performance. The lightGBM modeling is a novel technique that has been widely adopted in tumors survival prediction but not been widely adopted in critical care research [28, 29]. Otaguro et al. evaluated data from patients who underwent intubation for respiratory failure and received mechanical ventilation in ICU and use three learning algorithms (Random Forest, XGBoost, and LightGBM) to predict successful extubation, the result demonstrated that lightGBM exhibited the best overall performance [30]. Moreover, Yang et al. adopted nine machine learning models to predict in-hospital mortality in critically ill patients with hypertension and found that among nine machine learning models, the lightGBM model had the best predictive ability [31].

We employed visualization function in SHAP to find the effect of the specific value of each variable on model output. There are some factors contributing most including SOFA score, anion gap, albumin and so on. SOFA score is an useful tool to quantify the degree of organ dysfunction or failure present on ICU admission which has been widely used for in-hospital mortality prediction in the ICU settings [32–35]. And SOFA score was reported to exhibit better performance than other score systems in predicting infection-related in-hospital mortality in ICU patients, the higher the SOFA score, the higher the risk of in-hospital mortality [36]. Anion gap (AG) is commonly used to classify acid-base disorders and to diagnose various conditions. Recently, AG has been reported to associated with in-hospital mortality in ICU patients. Hu et al. indicated that AG was related to in-hospital mortality in intensive care patients with sepsis [37]. Moreover, Chen et al. demonstrated that AG could significantly predict ICU mortality for aortic aneurysm patients [38]. Hypoalbuminemia is almost associated with worse prognosis. And low albumin level was usually related to higher risk of in-hospital mortality in ICU settings [39]. Moreover, SHAP force plots of a dead and a survival patient (Fig 5) were selected to further verify the effect of features on the prediction ability and the results further confirmed the SOFA score, anion gap, albumin, etc. features have positive or negative effect on the output of these predictive models.

We should acknowledge some limitations of this research. First, the retrospective and observational nature of our study may lead to inevitable selection bias. Second, the data used in this study were based on public databases MIMIC-IV and eICU-CRD, an external validation is required to prevent overfitting. Third, the data did not include any information on the pathologic and radiologic finding of lung cancer. We could not differentiate between small cell carcinoma and non-small cell carcinoma, the algorithm model is skewed because important medical information about molecular diagnosis.

## Conclusions

In the present study, we applied six machine learning methods to predict in-hospital mortality in critically ill lung cancer patients. We demonstrated that the ensemble model achieved the best predictive performance and the lightGBM model exhibited the best performance for single model prediction. And the SOFA score, anion gap and albumin are the most important factors which impacted on the output of the machine learning models in predicting in-hospital mortality of critically ill patients with lung cancer. Our study obtained clinical feature interpretations to provide clinicians in ICU with some information for reference in clinical prognosis prediction.

## Declarations

### Ethics approval and consent to participate

The study was ethically approved by an affiliated of the Massachusetts Institute of Technology (No.27653720). All patients-related information in the database is anonymous, so there is no need to obtain the informed consent of the patients. This study is described in conformity to the Strengthening the Reporting of Observational Studies in Epidemiology (STROBE) statement, and was managed to conform to the tenets of the Declarations of Helsinki.

## Supporting information

S1 Fig The web-based training courses. (TIF)

S2 Fig The importance of all features derived from random forest in the validation set. (TIF)

S3 Fig The importance of all features derived from lightGBM in the validation set. (TIF)

S4 Fig The importance of all features derived from XGBoost in the validation set. (TIF)
