# Supplementary figures and images for: Machine learning for prediction of in-hospital mortality in lung cancer patients admitted to intensive care unit
Source: PLoS One. 2023 Jan 26;18(1):e0280606. doi: 10.1371/journal.pone.0280606 (PMC9879439; doi:10.1371/journal.pone.0280606)

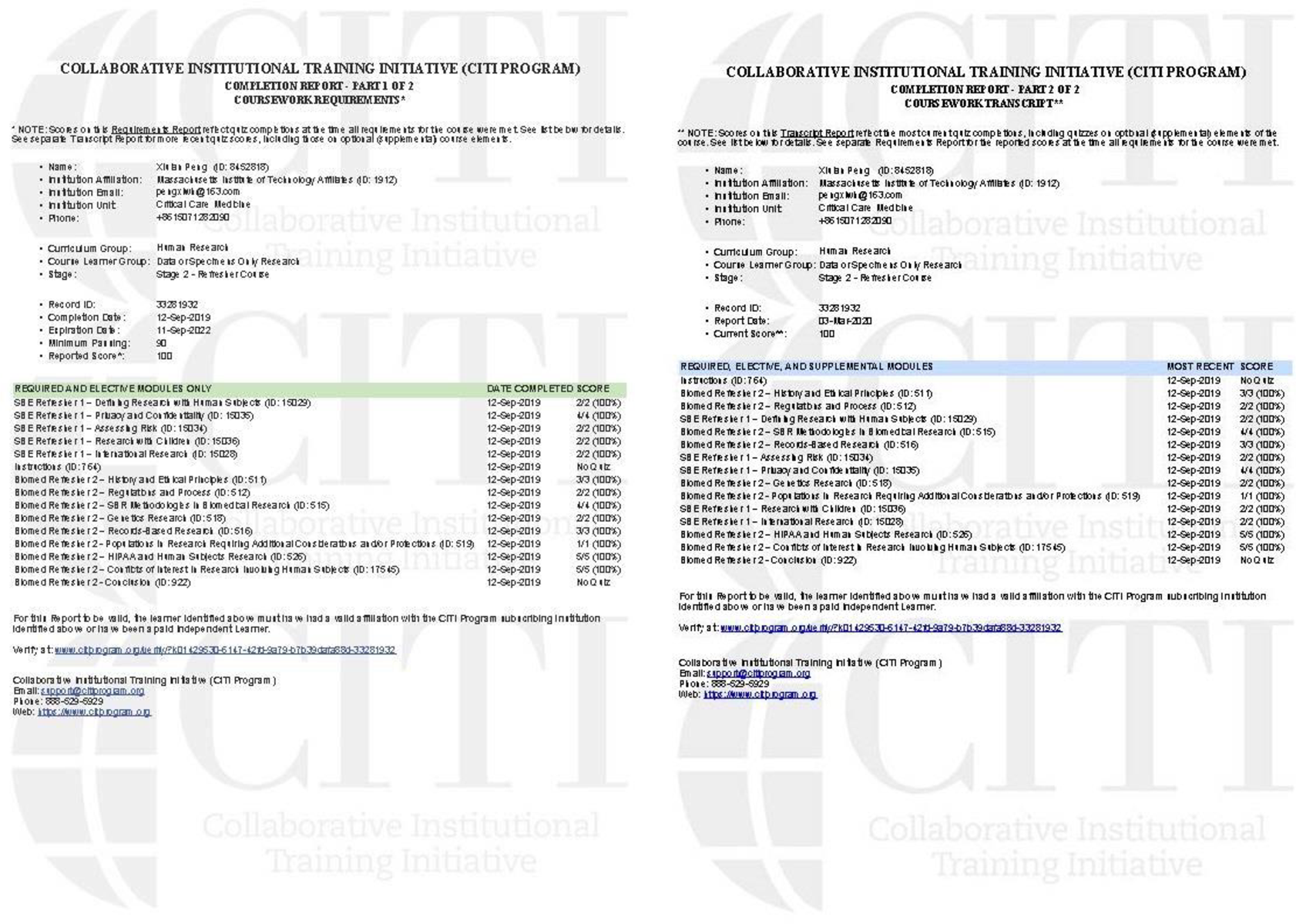

Supplement: S1 Fig — (TIF) [file pone.0280606.s001.tif]

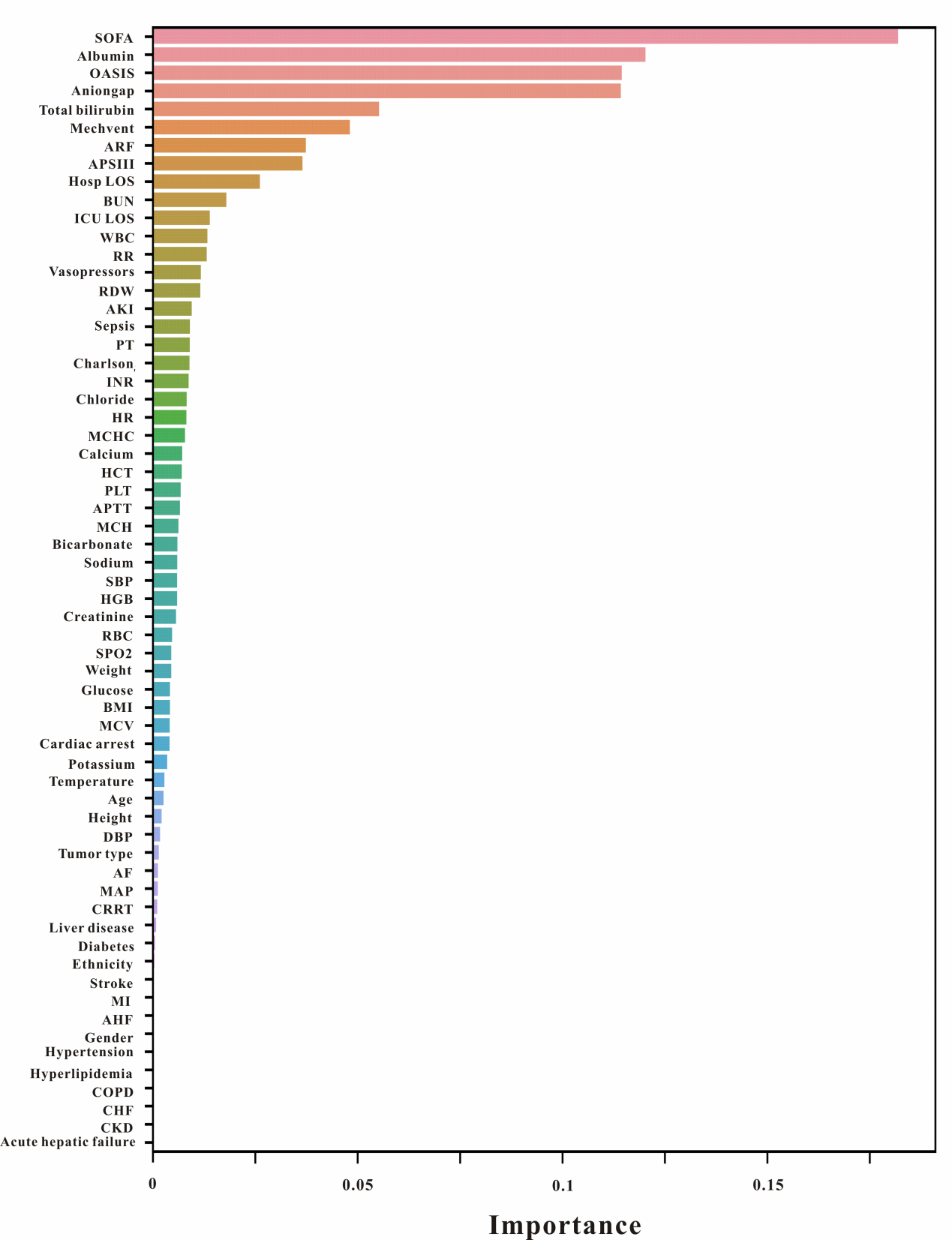

Supplement: S2 Fig — (TIF) [file pone.0280606.s002.tif]

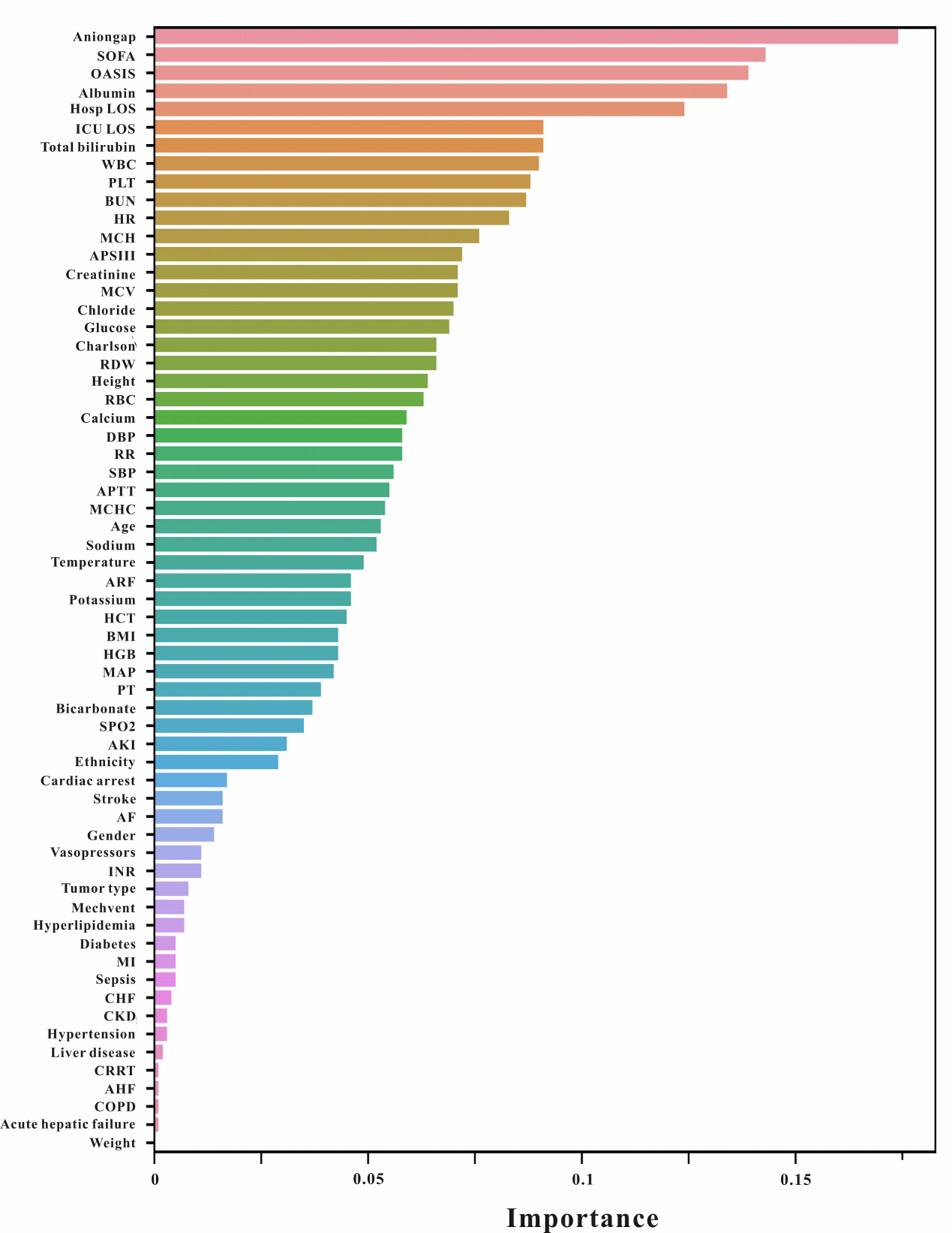

Supplement: S3 Fig — (TIF) [file pone.0280606.s003.tif]

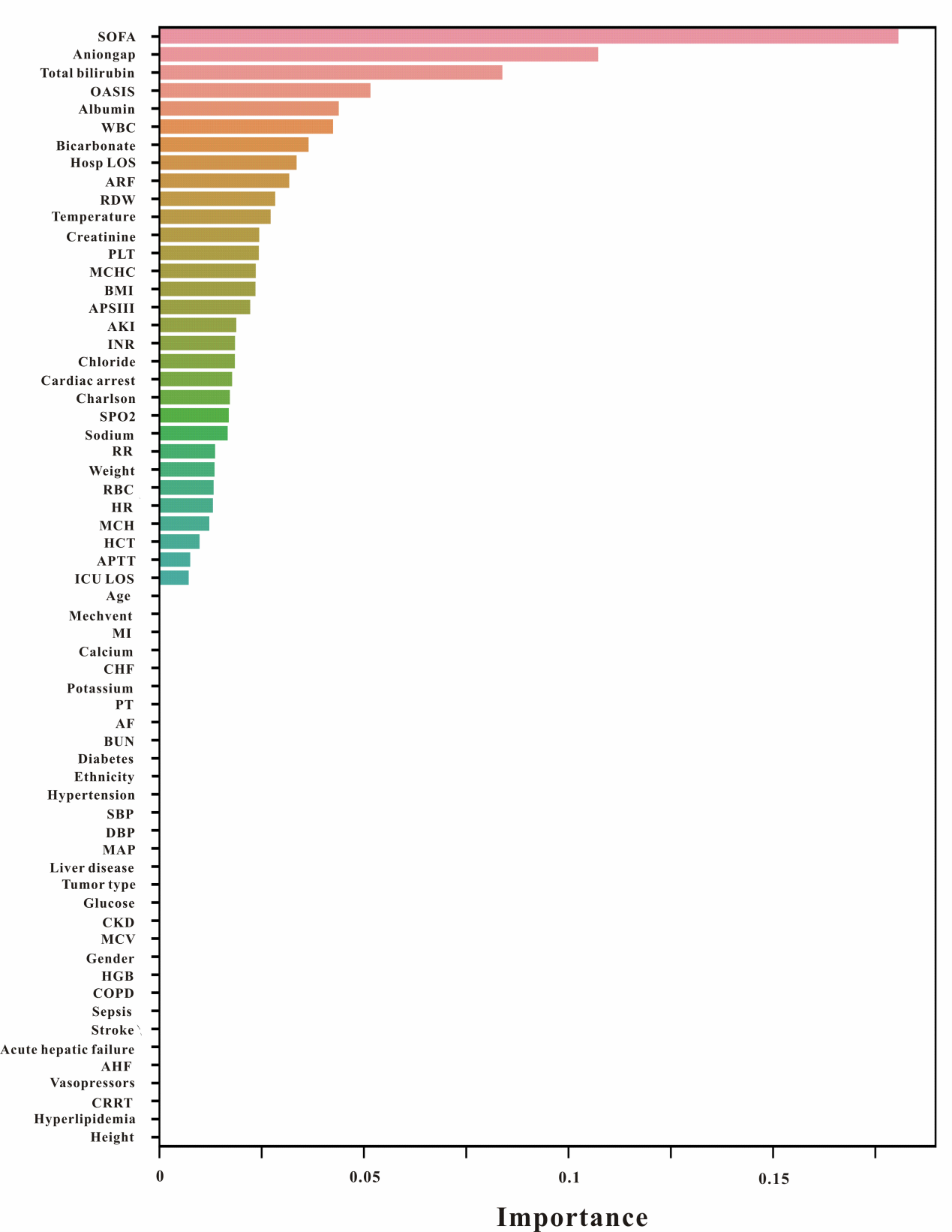

Supplement: S4 Fig — (TIF) [file pone.0280606.s004.tif]
